# Inhibition of HIV-1 replication by primer RNA packaging inhibitors

**DOI:** 10.1128/mbio.02087-25

**Published:** 2025-10-23

**Authors:** Marc Mirande, Frédéric Subra, Clémence Richetta, Eric Deprez, Olivier Delelis

**Affiliations:** 1 Laboratoire de Biologie et Pharmacologie Appliquée (LBPA), UMR 8113 CNRS, ENS Paris-Saclay, Université Paris-Saclay 27048, Gif-sur-Yvette, France; Dana-Farber Cancer Institute, Boston, Massachusetts, USA

**Keywords:** human immunodeficiency virus, integrase, host-pathogen interactions, lysyl-tRNA synthetase, lacidipine, azelastine

## Abstract

An essential step in the life cycle of human immunodeficiency virus type 1 (HIV-1) is the packaging of tRNA_3_^Lys^ during viral assembly. This step, which requires the assembly of a ternary complex consisting of GagPol, mitochondrial lysyl-tRNA synthetase, and tRNA_3_^Lys^, is essential for the production of infectious viruses. Indeed, it allows the initiation of reverse transcription in the particles. In this work, we used an *in vitro* assay to isolate molecules from a chemical library that disrupt the interaction between the integrase domain of GagPol and mitochondrial lysyl-tRNA synthetase. The three selected molecules also inhibit HIV-1 replication in an *ex vivo* assay. The number of HIV-1 particles produced in the presence of the drugs is greatly reduced, with a half-maximal inhibitory concentration obtained at a drug concentration of 5 µM. In addition, we have shown that the few particles produced in the presence of the drugs exhibit significantly reduced infectivity, due to their inability to synthesize minus-strand strong stop cDNA. In parallel, the tRNA_3_^Lys^ content of the particles obtained in the presence of drugs decreased significantly. The inability to package tRNA_3_^Lys^ into virions results in replication-defective particles.

**IMPORTANCE:**

Several families of inhibitors have been selected to tackle the human immunodeficiency virus (HIV). Cell entry, reverse transcriptase, integrase, or protease is the most popular targets along the viral life cycle. During the budding step of the virus, the packaging of tRNA_3_^Lys^ into particles, which serves as a primer for the initiation of reverse transcription in the virus, has been shown to be essential for the production of infectious virions. In this study, we isolated molecules that inhibit viral replication by inhibiting the incorporation of tRNA_3_^Lys^ into virions. The inhibitors of HIV-1 replication described in this work demonstrate that the HIV-1 tRNA packaging step can be exploited for the development of a new family of drugs with novel resistance profiles.

## INTRODUCTION

Human immunodeficiency virus type 1 (HIV-1) is a lentivirus belonging to the Retroviridae family that requires reverse transcription of its single-stranded RNA genome into a double-stranded cDNA that integrates into the genome of infected cells. To achieve the initiation step of reverse transcription into the nucleocapsid of the virus shortly after budding (1), the primer RNA must be packaged into the newly formed viral particles. This primer RNA is cellular tRNA_3_^Lys^ (2, 3). Human lysyl-tRNA synthetase (LysRS) has been shown to be involved in the recruitment of tRNA_3_^Lys^ into the virions through its interaction with Gag or GagPol (4–7). In humans, cytoplasmic and pre-mitochondrial LysRSs are encoded by the KARS1 gene through alternative splicing (8). The mature, active form of mitochondrial lysyl-tRNA synthetase (mLysRS) is produced in mitochondria after cleavage of its N-terminal mitochondrial targeting sequence (9). The catalytic domain of mLysRS interacts with the integrase (IN) domain of the GagPol polyprotein precursor to form the tRNA_3_^Lys^ packaging complex (10, 11). Another group previously proposed that the LysRS incorporated into HIV-1 originates from the cytosolic multisynthetase complex (MSC) (5). According to this model, after dissociation from the complex following phosphorylation at Ser207 by MAPK, cytosolic LysRS (cLysRS) would associate with Gag for packaging into the viral particles (12). The cytosolic and mature mitochondrial LysRSs have specific N-terminal sequences of 21 and 19 amino acid residues, respectively (9). Monospecific antibodies directed against these extra-peptides identified the human mLysRS species only in extracts of HIV-1 particles (6).

Effective inhibitors of HIV-1 replication have been described. They target different steps of the viral life cycle and include (i) co-receptor antagonists and fusion inhibitors that prevent cell infection (13), (ii) nucleoside analog reverse transcriptase inhibitors and non-nucleoside reverse transcriptase inhibitors that target the reverse transcription step (14), (iii) IN strand transfer inhibitors (INSTIs) that target the chromosomal integration step (15), (iv) allosteric IN inhibitors which block the IN-directed localization of viral RNA into the capsid (16), and (v) protease inhibitors that block the maturation step (17). Due to the high propensity for the emergence of resistance resulting from the high level of mutations accumulated during the reverse transcription step, multi-therapies are used to block viral replication (18). Since primer RNA incorporation into virions also controls viral infectivity (19), we hypothesized that blocking tRNA_3_^Lys^ incorporation into viral particles might be another means of controlling viral replication.

We have previously shown that the mitochondrial species of human lysyl-tRNA synthetase interacts with the GagPol polyprotein precursor of HIV-1 (10) and that the C-terminal IN domain of GagPol is the major contributor to this interaction (4). More specifically, the C-terminal domain (CTD) of IN is the major domain interacting with mLysRS. We first isolated molecules from a chemical library that inhibit the mLysRS:IN association *in vitro*. These compounds were then used in *ex vivo* assays to test their ability to inhibit HIV-1 replication. In this work, we have isolated three molecules that render HIV-1 particles defective for replication by decreasing the uptake of tRNA_3_^Lys^ into virions and inhibiting reverse transcription initiation. Our data provide strong proof of concept for the selection of the tRNA_3_^Lys^ packaging complex as a new operational target for HIV-1 replication inhibition.

## RESULTS

### Isolation of molecules inhibiting mLysRS:IN interaction

**Fig 1:**
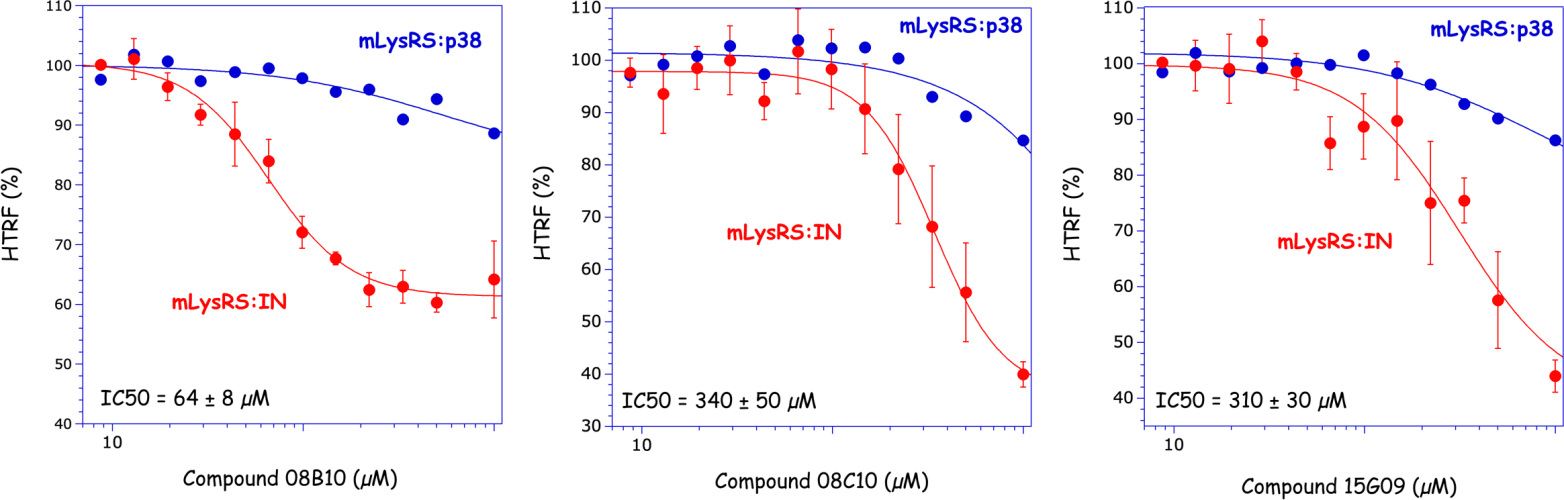
Determination of the half-maximal inhibitory concentration (IC50) of the drugs in the mLysRS:IN binding assay. The binding of mLysRS to IN (red) or p38 (blue) was monitored in the HTRF assay in the presence of increasing drug concentrations (9–1,000 µM). The experimental values (symbols) were fitted (curves) to the equation *Y* = *Y*min + {(*Y*max *− Y*min)/[1 + (*X*/IC50)*^n^*]}, where *n* is the Hill coefficient. The IC50 of the drugs in the mLysRS:IN binding assay and the associated standard deviations (*n* = 4) are shown.

High-throughput screening of a chemical library was performed using an HTRF assay developed to monitor the interaction between human mLysRS and HIV-1 IN (4). The “Prestwick Chemical Library” contains 1280 FDA-approved drugs. Three molecules were isolated for their ability to inhibit the interaction of mLysRS with IN (Fig. 1), but not with p38, another protein that does interact with LysRS *in vitro*, and associates cLysRS to the MSC *in vivo* (11). The half-maximal inhibitory concentration (IC50) in the mLysRS:IN assay ranged from 64 ± 8 µM for 08B10, 310 ± 30 µM for 15G09, to 340 ± 50 µM for 08C10. In this experiment, the background level of the HTRF signal (in %) is around 40%–50% of the value obtained in the absence of inhibitor. The potency of these inhibitors was also determined in the mLysRS:IN-CTD222 interaction and the mLysRS:Pol interaction. IN-CTD222 corresponds to the C-terminal β-barrel domain of IN identified as the domain of IN interacting with mLysRS (4), and the Pol polyprotein corresponds to the C-terminal moiety of the GagPol polyprotein precursor containing IN at the very C-terminal extremity, which associates with mLysRS and tRNA^Lys^ to form the tRNA-packaging complex of HIV-1. As shown in Fig. S1, the three selected molecules also impede these interactions with similar IC50. This suggested that the three inhibitors might also be able to challenge the interaction between mLysRS and GagPol, the species of IN present at the budding stage of HIV-1 infection.

**Fig 2:**
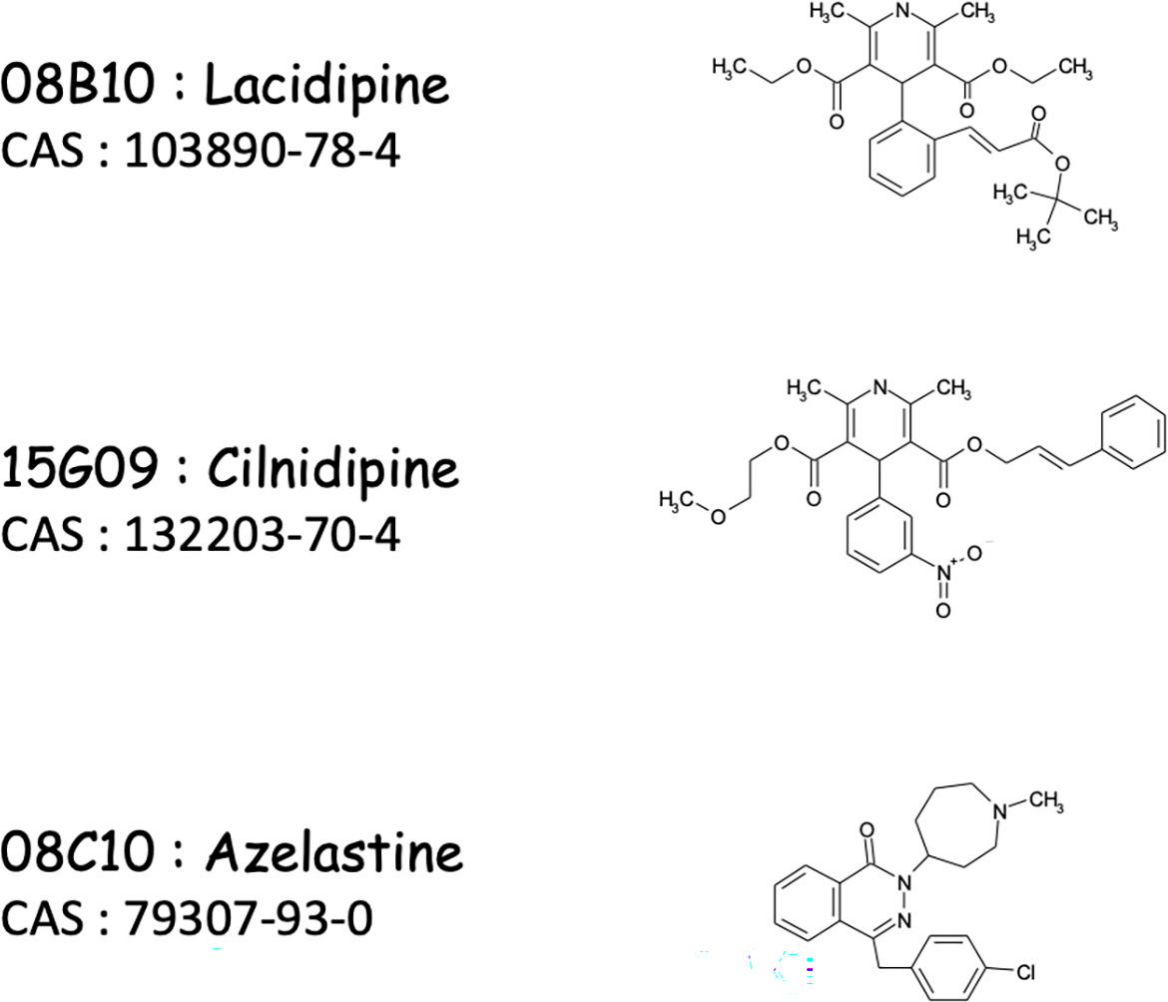
The chemical structure of the inhibitors. The library number, name, Chemical Abstracts Service (CAS) number, and chemical structure of the three selected inhibitors (patent application number WO 2022/084259 A1) are shown.

08B10 and 15G09 are lacidipine and cilnidipine (Fig. 2), respectively, and are built around a dihydropyridine ring. They have been reported as calcium channel blockers and display antihypertensive properties (20). 08C10 is azelastine (Fig. 2), a benzyl phthalazone derivative with antihistaminic activity (21).

A number of dihydropyridine derivatives have been described (20). In a search for compounds more potent than lacidipine or cilnidipine, 18 dihydropyridine derivatives were assayed. Diludine, nimodipine, nifedipine, felodipine, celvidipine, or levamlodipine did not significantly alter the mLysRS:IN interaction in the HTRF assay. Amlodipine, nicardipine, isradipine, nitrendipine, or nivaldipine inhibit the interaction with an IC50 greater than 500 µM. Lercanidipine, azelnidipine, nisoldipine, benidipine, and manidipine display inhibitory profiles similar to lacidipine and cilnidipine (Fig. S2). These compounds preferably carry a bulky, cyclic, or branched group appended to positions R1 or R2.

### Inhibition of HIV-1 replication

According to our model, tRNA_3_^Lys^ packaging requires the assembly of a ternary complex consisting of GagPol, mLysRS, and tRNA_3_^Lys^. Thus, disruption of the GagPol:mLysRS interaction is expected to impair tRNA_3_^Lys^ packaging into the virus, resulting in a defective HIV-1 replication step due to the absence of the primer RNA required for initiation of reverse transcription.

**Fig 3:**
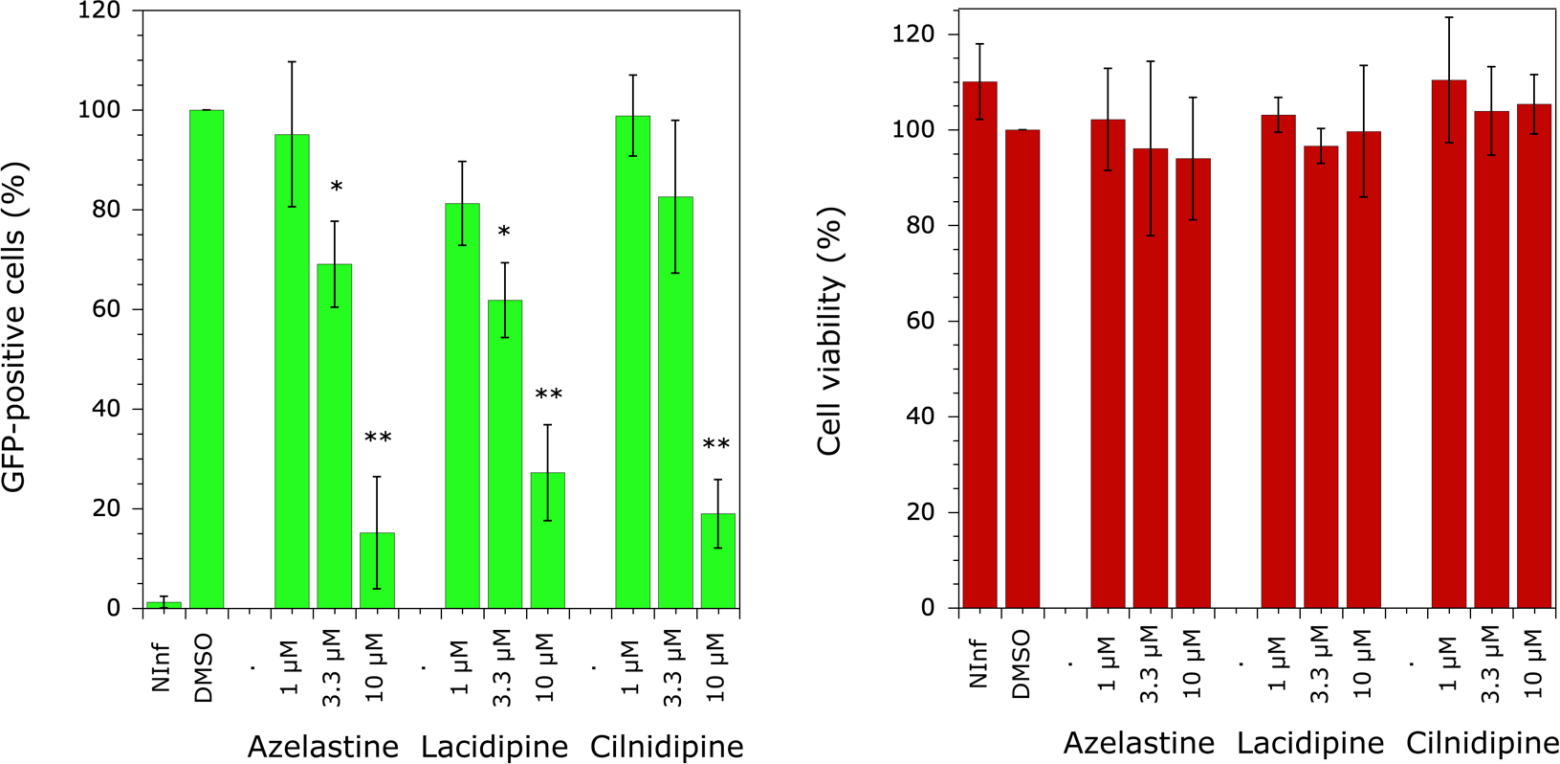
Effect of the inhibitors on HIV-1 replication and cell toxicity. MT4 cells were infected with NL4-3-GFP virus. Two days after infection, inhibitors were added to the culture medium at final concentrations of 1, 3.3, or 10 µM. The final concentration of DMSO was 0.1%. Five days after infection, GFP expression was quantified by flow cytometry, and cell viability was determined by the MTT assay. NInf corresponds to non-infected cells. Six independent experiments were performed, and standard deviations are shown. A Student *t*-test was used to determine statistical significance compared with the control experiment with DMSO (*: *P* < 0.001; **: *P* < 0.0001).

To test this hypothesis, azelastine, lacidipine, and cilnidipine were used in an *ex vivo* assay to determine their effect on HIV-1 proliferation. MT4 cells were infected with NLENG1-IRES-GFP, a virus that allows for multiple cycles of infection, and the molecules were added to the medium 36–48 h after infection, before the completion of the first round of viral infection; 72 h after the drugs were added, when two rounds of HIV-1 infection were underway, the level of GFP fluorescence was determined by flow cytometry, as a marker of HIV-1 infection (Fig. 3, left panel). HIV-1 replication was inhibited in a dose-dependent manner. When drugs were added at a final concentration of 10 µM, GFP fluorescence was reduced to 15.2%, 27.2%, or 18.9% of the value observed when DMSO was added alone for azelastine, lacidipine, and cilnidipine, respectively (Fig. 3, left panel). The half-maximal inhibitory concentration was obtained for a drug concentration of approximately 5 µM. Furthermore, although the number of GFP-positive cells is reduced, the intensity of GFP fluorescence is not affected, with a maximum fluorescence intensity of approximately 10^4^ units (Fig. S4). This indicates that viral DNA integration into the host genome is not affected, suggesting that replication does not proceed without integration, as observed in the case of HIV resistance to some INSTI drugs (22, 23). At the same time, no drug toxicity was observed, as determined by the MTT assay (Fig. 3, right panel). These data clearly demonstrate that the three selected molecules inhibit HIV-1 replication and are not toxic to cells. The selectivity index (SI) (24) of the three drugs, defined as the ratio of 50% cytotoxic concentration (CC50; >20 µM) to 50% inhibitory concentration (IC50; ~5 µM), >4.

**Fig 4:**
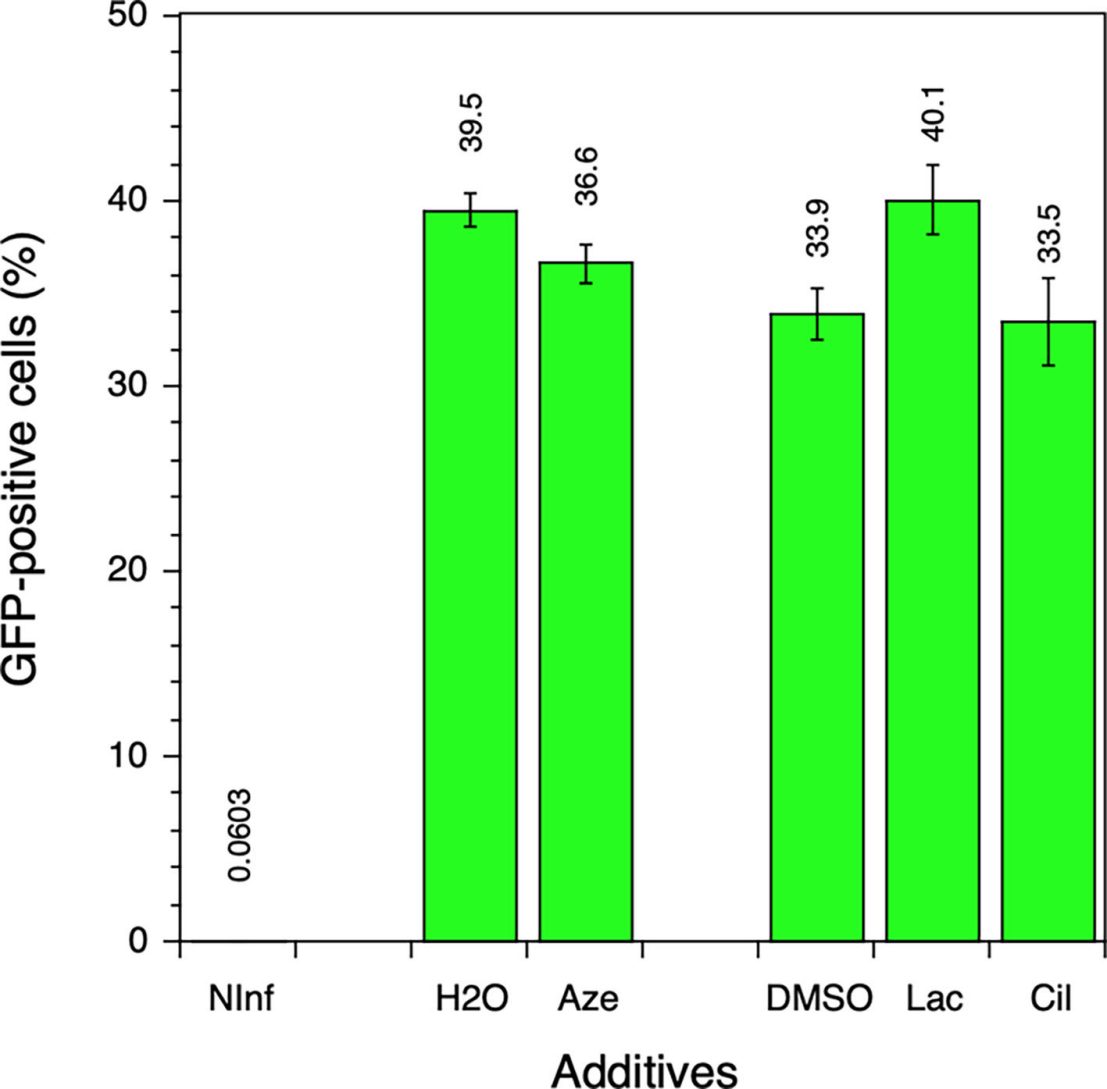
Effect of the inhibitors on a single cycle of HIV-1 replication. MT4 cells were infected with NLENG1-ES-IRES-GFP virus, and inhibitors were added to the culture medium immediately after infection at a final concentration of 10 µM. The final concentration of DMSO was 0.1% for lacidipine and cilnidipine. Two days after infection, GFP expression was quantified by flow cytometry. NInf corresponds to non-infected cells. Aze is azelastine, Lac is lacidipine, and Cil is cilnidipine. Values and the associated standard deviations (*n* = 3) are shown.

We verified that the three molecules did not interfere with the initial stages of HIV-1 replication. Using envelope-defective viruses that are only capable of a single cycle of infection, we observed that none of the three molecules inhibited the first round of replication, as shown by the level of GFP expression in MT4 cells infected with NLENG1-ES-IRES-GFP viruses (Fig. 4).

These drugs also did not inhibit the *in vitro* aminoacylation activity of LysRS. When added up to 1 mM, azelastine and cilnidipine did not inhibit Lysyl-tRNA^Lys^ synthesis, and lacidipine resulted in a 30% inhibition (Fig. S5).

Of the five other dihydropyridine derivatives that showed IC50s greater than 500 µM i*n vitro* (Fig. S2), nisoldipine did not inhibit HIV-1 replication in the *ex vivo* assay, lercanidipine and azelnidipine had higher cellular toxicity, and benidipine and manidipine were similar to lacidipine and cilnidipine (Fig. S3a and b). They were not used for the remainder of the study.

**Fig 5:**
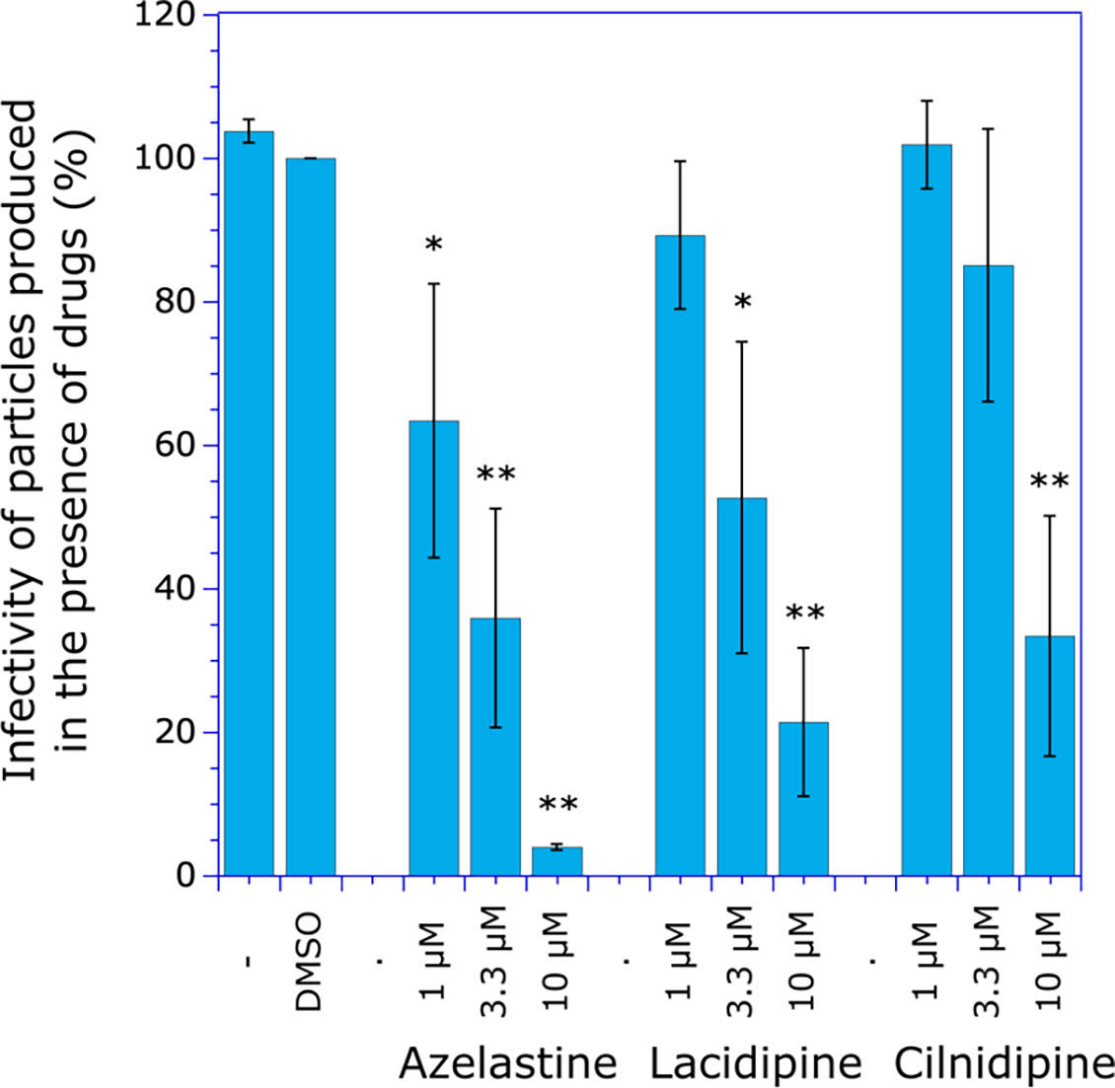
Infectivity of newly formed HIV-1 particles produced in the presence of drugs. MT4 cells were infected with the NL4-3 virus. Two days after infection, H_2_O (–), DMSO, or drugs diluted in H_2_O (for azelastine) or DMSO (for lacidipine and cilnidipine) were added to the culture medium at the final concentrations indicated. The final concentration of DMSO was 0.1%. After 24 h of incubation in the presence of drugs, viruses recovered in the culture supernatant were used to infect HeLa-P4 cells. The infectivity of HIV-1 viruses was determined by the CPRG method. Six independent experiments were performed, and standard deviations are shown. A Student *t*-test was used to determine statistical significance compared with the control experiment with DMSO (*: *P* < 0.001; **: *P* < 0.0001).

We used the CPRG method developed in HeLa P4 cells grown in 96-well plates to measure the infectivity of virus particles, as low levels of virus were recovered after exposure of infected MT4 cells to different drug concentrations. In this assay, expression of the LacZ gene is under the control of the HIV-1 LTR, and its level of trans-activation correlates with the level of expression of HIV-1 Tat. HeLa P4 cells were incubated with normalized amounts of virus as measured by the P24 assay, and the expression of β-galactosidase was determined by the CPRG assay (Fig. 5). As expected for viruses depleted in the tRNA_3_^Lys^ primer and consistent with the data of Fig. 3, viruses recovered after MT4 cells were exposed to 10 µM drugs exhibited low infectivity: less than 4%, 22%, and 34% for azelastine, lacidipine, and cilnidipine, respectively, compared with wild-type viruses.

### Effect of inhibitors on the synthesis of minus-strand strong-stop viral DNA

**Fig 6:**
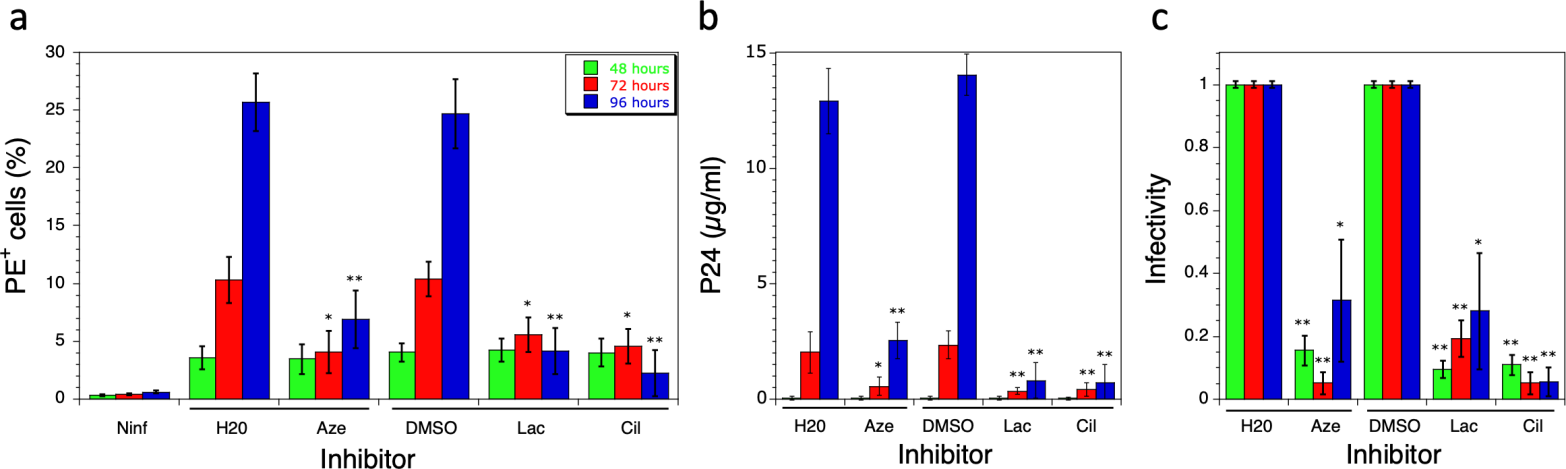
Effect of drugs on the first steps of the viral cycle. MT4 cells were infected with the NL4-3 virus and incubated for 48, 72, or 96 h in the presence of 10 µM drugs. For incubations of 72 h or 96 h, drugs were also added after 30 h or 48 h of incubation, respectively. (**a**) Gag expression was monitored by flow cytometry in non-infected cells (Ninf), or after the addition of 10 µM of azelastine (Aze), lacidipine (Lac), or cilnidipine (Cil), or H_2_O or DMSO as controls. The percentage of phycoerythrin-positive (PE^+^) cells, labeled with anti-Gag KC57-RD1 antibody, is shown. Values obtained at 48 h (green), 72 h (red), and 96 h (blue) are shown. (**b**) The amount of virus recovered at 48, 72, or 96 h was quantified by the P24 assay. (**c**) The infectivity of viruses recovered at 48, 72, or 96 h was determined by the CPRG assay. An infectivity of 1 corresponds to viruses recovered after incubation with H_2_O (control of azelastine) or with DMSO (control of lacidipine and cilnidipine). Three independent experiments were performed, and standard deviations are shown. A Student *t*-test was used to determine statistical significance compared with the control experiment with H_2_O or DMSO (*: *P* < 0.001; **: *P* < 0.0001).

Inhibition of HIV-1 replication following exposure of infected cells to 10 µM of the drugs was determined after infection of MT4 cells with the NL4-3 virus, and treatment of infected cells with trypsin to eliminate viruses remaining in solution, which could contaminate *de novo* produced particles recovered after different times of infection. Drugs were added 4 h after infection (Fig. 6); 48, 72, or 96 h after infection, the extent of HIV-1 replication was monitored by quantifying the expression of Gag after incubation with a fluorescently labeled IgG (KC57-RD1) and FACS analysis (Fig. 6a). The level of Gag expression after 48 h of infection was not affected by the presence of drugs. By contrast, the effect of drugs on multi-cycle virus replication is clearly visible after 72 or 96 h of infection, when expression of Gag is reduced by 80% compared with controls (Fig. 6a). This is consistent with a mechanism of inhibition affecting the late steps of HIV replication that would limit tRNA_3_^Lys^ packaging, resulting in particles that are unable to initiate reverse transcription and are thus deficient for the second round of infection.

The amount of virus released into the culture medium after 48, 72, or 96 h of HIV-1 infection was quantified after concentration by ultracentrifugation. After 72 or 96 h of infection in the presence of drugs, only 10%–20% of viruses were recovered, compared with controls (Fig. 6b), consistent with the lowest Gag expression observed above. The infectivity of these viruses was determined by the CPRG assay after incubation of HeLaP4 cells with normalized amounts of virus as measured by the P24 assay. After 48 h of infection in the presence of drugs, the few viruses recovered after ultracentrifugation showed an infectivity reduced by more than 80% (Fig. 6c). The infectivity of viruses recovered after 72 or 96 h of infection remained low. Thus, when MT4 cells infected with NL4-3 viruses are incubated in the presence of any of these three inhibitors, the amount of virus produced is greatly reduced (Fig. 6b), and their infectivity is impaired (Fig. 6c). Taken together, these results show that the molecules reduce the infectivity of newly formed viruses. This results in reduced virus production after two or more cycles of replication.

**Fig 7:**
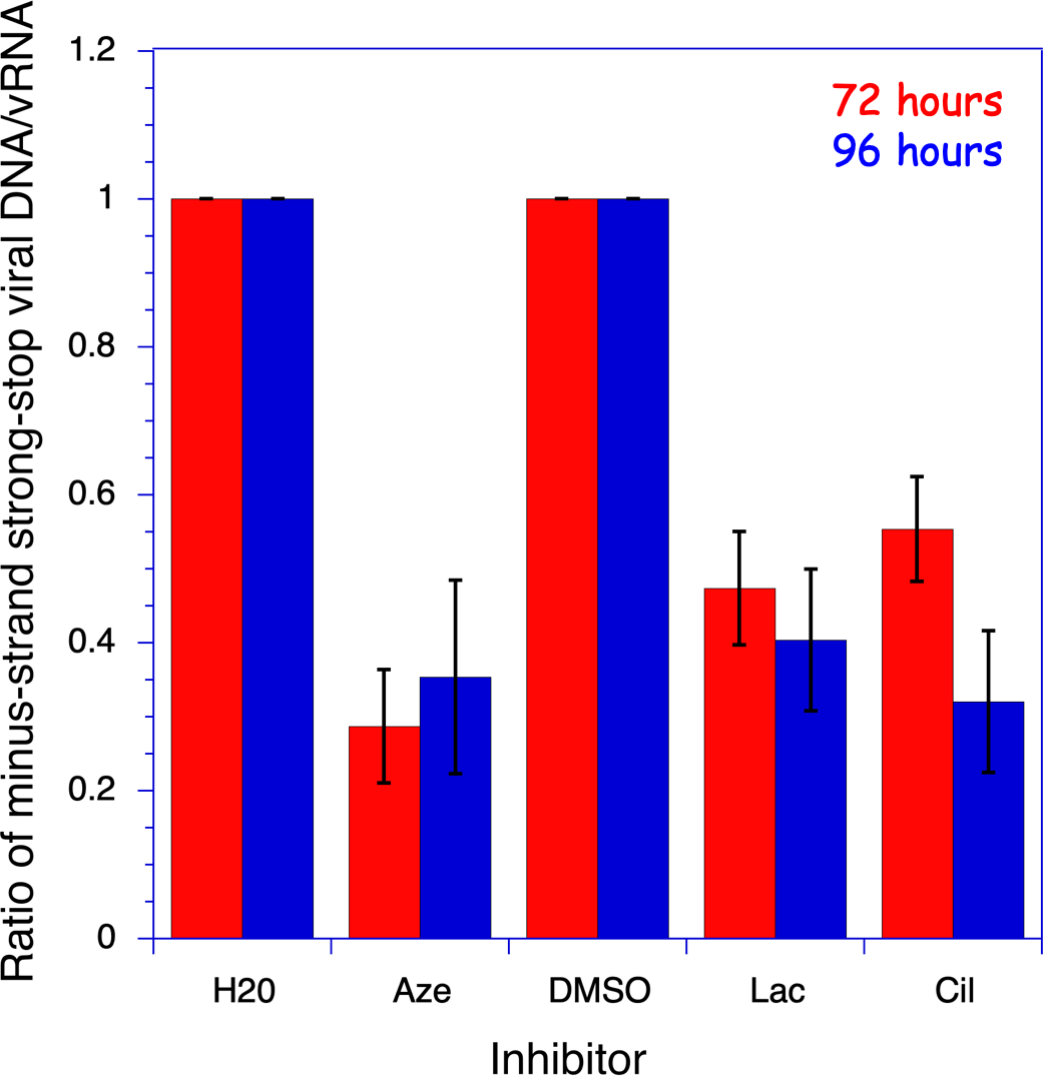
Effect of drugs on viral synthesis of minus-strand strong stop cDNA. Viruses were harvested from the culture medium after 72 (red) or 96 h (blue) of infection of MT4 cells with NL4-3 viruses. Minus-strand strong stop viral DNA and viral RNA (vRNA) were quantified by digital PCR and RT-dPCR, respectively. Viruses were harvested from cultures grown in the presence of 10 µM of azelastine (Aze), lacidipine (Lac), or cilnidipine (Cil), or H_2_O or DMSO as controls. Values and the associated standard deviations (*n* = 3) are expressed as the ratios of the values obtained for the H_2_O or DMSO controls. A *P* value < 0.001 was obtained in a Student *t*-test for samples with azelastine, lacidipine, or cilnidipine.

We used digital RT-PCR and digital PCR to determine the viral mRNA and minus-strand strong stop viral cDNA content of the particles (Fig. 7; Fig. S6). The amount of early viral cDNA produced in the HIV-1 particles is a measure of the efficiency of the initiation step of reverse transcription, which requires the packaging of tRNA_3_^Lys^ at the budding step, the primer for cDNA synthesis.

After 48 h of infection, corresponding to a single replication cycle, the number of viral particles produced was very low (see P24, Fig. 6b), precluding accurate quantification of viral DNA and RNA. In contrast, viral DNA and RNA could be nicely quantified by digital PCR after 72 or 96 h of infection (Fig. S6). The amount of minus-strand strong stop cDNA present in the viruses, normalized to the amount of viral RNA present, was significantly reduced 72 and 96 h after infection in the presence of 10 µM of either azelastine, lacidipine, or cilnidipine (Fig. 7). Thus, the ability of viral particles to promote the reverse transcription initiation step is altered in the presence of these drugs, as expected for tRNA_3_^Lys^-deficient viral particles. The low levels of minus-strand strong stop cDNA detected in the viruses produced in the presence of the drugs explain the low infectivity of these particles.

### tRNA_3_^Lys^ deficiency in viruses obtained after exposure to inhibitors

**Fig 8:**
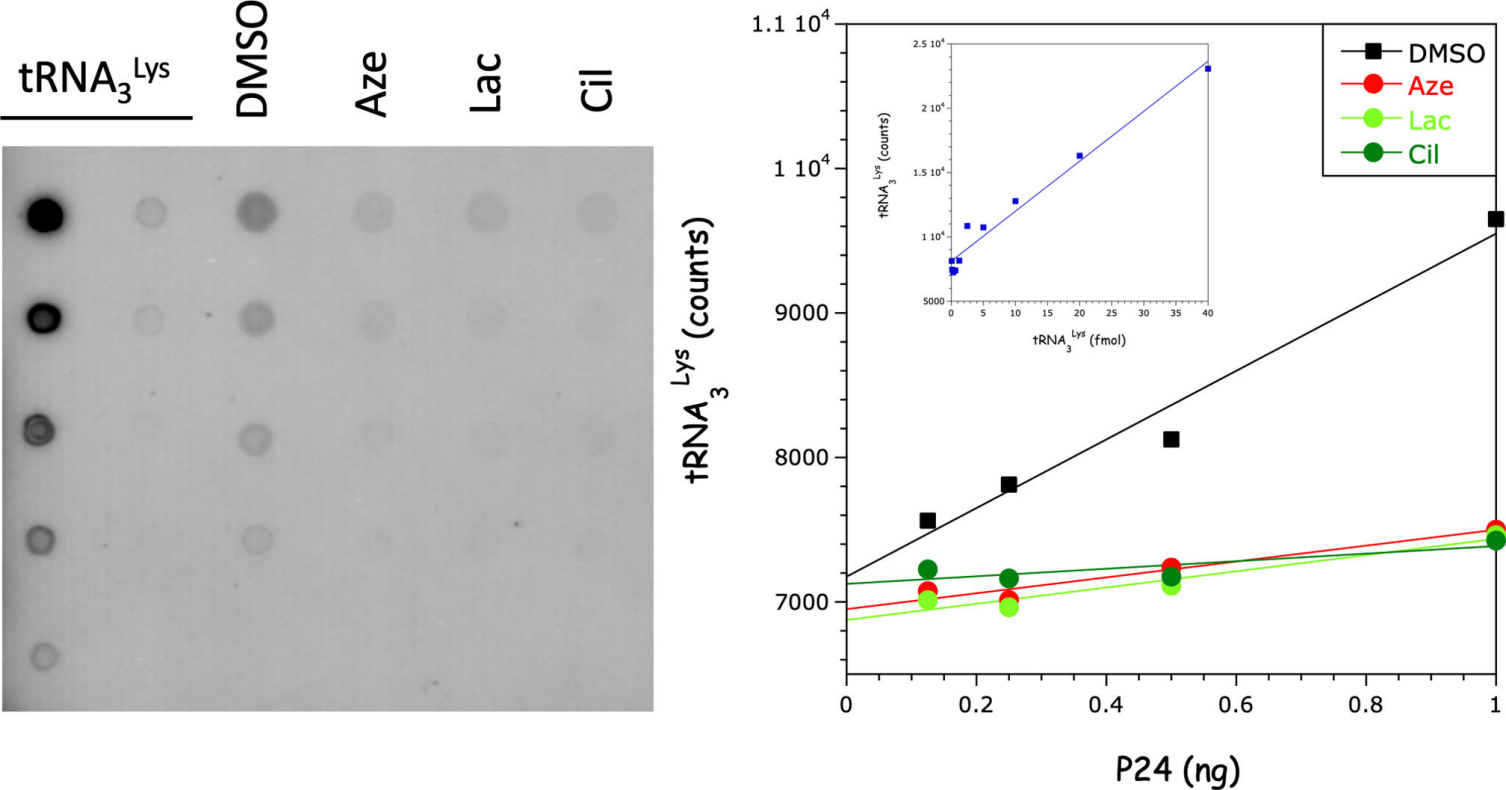
Effect of drugs on tRNA_3_^Lys^ uptake into viral particles. tRNA was extracted from viruses recovered in the supernatant of cultures grown in the presence of 10 µM of azelastine (Aze), lacidipine (Lac), or cilnidipine (Cil), or dimethyl sulfoxide (DMSO) as a control. The samples were serially diluted and spotted onto a nitrocellulose filter, corresponding to 1.0, 0.5, 0.25, and 0.125 ng of P24. tRNA_3_^Lys^, ranging from 40 to 0.315 fmol, was spotted as an internal control. After hybridization with a ^32^P-labeled oligonucleotide probe, the radioactivity was quantified using a Typhoon and plotted as a function of the P24 equivalent amount in the samples. Inset: standard curve obtained with pure tRNA_3_^Lys^. This experiment was performed twice.

MT4 cells were infected with the NL4-3 virus. After 72 h, the cells were exposed to 10 µM of azelastine, lacidipine, or cilnidipine. The viruses were then recovered from the cell culture supernatant and purified by centrifugation through a sucrose cushion. Recovery of the viruses was quantified using the P24 assay. The tRNA was extracted with phenol-ether and precipitated with ethanol. Samples corresponding to 1, 0.5, 0.25, and 0.125 ng of P24 were spotted onto nitrocellulose membranes and hybridized with a radiolabeled oligonucleotide probe complementary to tRNA_3_^Lys^ (Fig. 8). The amount of tRNA_3_^Lys^ was significantly reduced in the samples obtained after incubation with the drugs, compared with the DMSO control. Quantification using pure tRNA_3_^Lys^ as a control showed the presence of 10–15 tRNA molecules per viral particle. In samples obtained in the presence of drugs, the tRNA content was reduced 4-fold to 5-fold.

## DISCUSSION

We have previously shown that the human mitochondrial species of lysyl-tRNA synthetase (mLysRS) is hijacked by the virus during HIV-1 particle assembly (6). It interacts with the Pol domain of the GagPol polyprotein precursor (10) and more specifically with the C-terminal β-barrel domain of IN (4) to form the tRNA_3_^Lys^ packaging complex (11). In this work, we have isolated inhibitors of the interaction between human mLysRS and HIV-1 IN. These molecules, which were selected *in vitro* after screening of a chemical library for their ability to inhibit the interaction between the two purified proteins, were also shown to efficiently block HIV-1 replication in an *ex vivo* assay. The half-maximal inhibitory concentration (IC50) of these molecules was estimated to be around 50–300 µM in the *in vitro* assay (Fig. 1). However, due to the poor solubility of IN, which can form multiple oligomers (25), we suspected that these values were underestimated. Indeed, when these molecules were used in an *ex vivo* assay, they showed an operational IC50 of about 5 µM and did not elicit cell toxicity (Fig. 3). Two of these molecules, lacidipine and cilnidipine, have a dihydropyridine backbone, but none of the other 16 derivatives tested showed a better inhibitory profile in the *in vitro* and *ex vivo* assays (Fig. S2 and S3).

The molecules isolated in this work do not inhibit LysRS tRNA-aminoacylation activity, nor do they inhibit the first stages of HIV-1 infection, from fusion to integration. However, new HIV-1 particles formed and released in the presence of the drugs are replication-defective. We used quantitative PCR to analyze the RNA and DNA content of the viruses exposed to the drugs. In particular, the viral content of minus-strand strong stop cDNA is greatly reduced, whereas the viral RNA content is unaffected, indicating a defective reverse transcription initiation mechanism within the particles. The number of particles produced in the presence of drugs is significantly reduced (Fig. 6b), making it challenging to obtain a sufficient amount of virus for the precise quantification of tRNA_3_^Lys^ in these particles. Nevertheless, the tRNA_3_^Lys^ content appears to be substantially diminished (Fig. 8). These data are consistent with a reduced level of tRNA_3_^Lys^ packaging. These results show that the minus-strand strong stop cDNA is present in newly formed particles, but not after treatment with drugs. This strongly supports the idea that reverse transcription begins in newly made virions before they infect new cells.

The three molecules isolated and characterized here clearly demonstrate that the HIV-1 tRNA packaging step can be targeted by a new family of drugs with novel resistance profiles. Our data provide strong proof of concept for the use of the tRNA_3_^Lys^ packaging complex as a novel target for HIV-1 replication inhibition.

## MATERIALS AND METHODS

### HTRF assay

Homogeneous time-resolved fluorescence (HTRF) assays were performed in black, flat-bottom, half-area, 96-well microplates (Corning #3694). Proteins were isolated as described previously (4). Several batches of IN were used throughout this study. It was determined that IN, IN-CTD222, and Pol proteins were greater than 90% homogeneous. Human mLysRS with a C-terminal HA-tag (mLysRS-HA) was incubated at a dimer concentration of 1.5 nM with HIV-1 IN carrying a C-terminal His-tag at a dimer concentration of 25 nM, in 10 mM Tris-HCl pH 7.5, 50 mM NaCl, 10 mM 2-mercaptoethanol, and BSA at 1 mg/mL, in the presence of 0.2 mM of the molecules of the library (Prestwick Chemical Library, 1280 compounds) (https://www.prestwickchemical.com/screening-libraries/prestwick-chemical-library/). The molecules are dissolved in DMSO at a final concentration of 10 mM. After a 1 h incubation on ice, antibodies (Cisbio) directed to the His-tag and conjugated with Eu^3+^ cryptate (Cisbio #61HISKLB) and the HA-tag conjugated with XL665 (Cisbio #610HAXLB) were added, and incubation was continued for 30 min. After the addition of 50 mM KF, fluorescence of Eu^3+^ cryptate and of XL665 was recorded at 620 nm (*I*_620_) and 665 nm (*I*_665_), respectively, following excitation of Eu^3+^ cryptate at 317 nm, in an Infinite M1000 PRO microplate reader (TECAN). The results were expressed as the ratio of *I*_665_/*I*_620_.

### Cells and viruses

MT4 cell line (26) was maintained in RPMI 1640. HEK293T and HeLa-P4 cells (27) were maintained in Dulbecco’s modified Eagle medium (DMEM). All media were supplemented with Glutamax and with 10% heat-inactivated fetal calf serum (Hyclone) and 1% penicillin/streptomycin (100 units/mL) (Gibco). All media were purchased from Gibco (Life Technologies Co.). All cell lines used here were incubated at 37  °C, under 5% CO_2_ atmosphere. HIV-1 stocks were prepared by calcium phosphate-mediated transfection of HEK293T cells, as previously described (22), with shuttle vector plasmids encoding HIV-1 NL4-3 (GenBank: AF324493.1) or NLENG1-IRES-GFP (28). The latter vector comes from the HIV NL4-3 strain and contains a *gfp-*IRES-*nef* cassette at the *nef* locus. For clarity reasons, we designate this vector here as “NL4-3-GFP.” For single-round infection assays, the envelope-defective NLENG1-ES-IRES-GFP virus, pseudotyped with VSV-G protein, was used.

The HIV-1 p24^gag^ antigen contents in viral inoculates were determined by enzyme-linked immunosorbent assay (Perkin-Elmer Life Sciences).

### HIV infectivity and toxicity assays

Replication of the NL4-3 virus was determined either by the ELISA technique or in HeLa-P4 cells by the CPRG method as described previously (29). These are HeLa CD4 LTR-lacZ cells in which the expression of lacZ is induced by the HIV transactivator Tat, allowing precise quantification of the infectivity of HIV-1. The viral titer was determined by quantifying β-galactosidase activity in HeLa-P4 lysates in a colorimetric assay based on the cleavage of chlorophenol red-β-D-galactopyranoside (CPRG) by β-galactosidase. The cytotoxicity is evaluated by the MTT assay.

For NL4-3-GFP vectors, viral infection is followed by GFP expression (i.e., the percentage and geometric mean fluorescence intensity [MFI] of GFP^+^ cells). Infectivity was estimated by flow cytometry using a FACS Celesta flow cytometer (BD Biosciences). Toxicity is also assessed by flow cytometry (side and forward scatter).

For NL4-3 vectors, viral replication is followed by intracellular Gag staining with anti-gag antibody KC57-FITC or KC57-RD1 (Beckman Coulter), as described (22).

### Quantification of viral RNA and DNA

MT4 cells (2 × 10^6^ cells) were infected with the NL4-3 viruses. After 4–5 h of infection at 37°C, cells were twice washed with PBS, incubated 5 min at 37°C in 0.25% trypsin-EDTA solution (Gibco) to eliminate viruses remaining in the medium, incubated 5 min at 37°C in complete RPMI medium, twice washed with PBS, resuspended in RPMI medium at a cell density of 200 × 10^3^ cells/mL and incubated with 10 µM of azelastine (25 mM stock solution in H_2_0), or lacidipine and cilnidipine (25 mM stock solution in DMSO). A second aliquot of the inhibitors was added to the culture medium 48 h after infection. Cultures were collected 48, 72, or 96 h after infection; the cells and cellular debris were removed by centrifugation; and the supernatant was subjected to high-speed centrifugation (SW41 rotor, 20,000 rpm, 2 h at 10°C) to pellet the viruses. Viruses were resuspended in 200 µL of PBS, and 50 µL samples were mixed with a pellet containing 500 × 10^3^ uninfected MT4 cells to serve as RNA and DNA carriers. Viral RNA and DNA were purified with the RNeasy minikit (Qiagen) and QIAamp DNA blood minikit (Qiagen), respectively, according to the manufacturer’s instructions. All RNA and DNA quantifications were performed by digital PCR and RT-qPCR on a QIAcuity instrument (QIAGEN) using 26K 24-well nanoplates and the QIAcuity Probe PCR and One-Step Viral RT-PCR kits. Primers and probes were 5′-ATCTGAGCCTGGGAGCTCTCT, 5′-CTGCTAGAGATTTTCCACACTGAC, and 5′-FAMAAGTAGTGTGTGCCC for the quantification of minus-strand strong-stop cDNA, and 5′-CTGAAGCGCGCACGGCAA, 5′-GACGCTCTCGCACCCATCTC, and 5′-FAMTAGCCTCCGCTAGTCAAAATTTTTGG CGT for viral RNA quantification.

### Quantification of tRNA_3_^Lys^

MT4 cells were infected with NL4-3 viruses (200  ng of p24 antigen per 10^6^ cells). Twenty-four hours after infection at 37°C, the cells were washed three times with PBS and resuspended in RPMI medium at a cell density of 500 × 10^3^ cells/ml, and plated in six-well plates (4 mL per well) in the presence of inhibitors at a final concentration of 10 µM. The concentration of DMSO in the cultures is 0.1%. Seventy-two hours post-infection, the cells were centrifuged for 5 min at 400 × *g* and 12°C. The supernatant was then subjected to a second centrifugation step for 20 min at 1,300 × *g* and 12°C to remove cell debris. The clear supernatant was subjected to ultracentrifugation on a 20% sucrose cushion (2.5 h at 20,000 rpm in a SW55Ti rotor, at 12°C). The virus pellets were resuspended in 100 µL of PBS and stored at −80°C. The amount of virus was determined by the P24 assay.

After two phenol-ether extractions, the tRNA was ethanol-precipitated, and the resulting pellet was dissolved in 20 µL of H_2_O. The samples were serially diluted in H_2_O, and 5 µL of each dilution was spotted onto Nytran membranes (Nytran 0.45, Schleicher & Schuell). The tRNAs were fixed with a three-minute UV exposure. Pure *in vitro*-transcribed tRNA_3_^Lys^ (23) was used as an internal standard. The membrane was hybridized with an oligonucleotide probe (5'-cctggaccctcagattaaaagtctga-3') radiolabeled using T4-polynucleotide kinase (Roche) and [γ-^32^P]-ATP. Radioactivity was quantified using a Typhoon (GE Healthcare).

### tRNA aminoacylation assay

Initial rates of tRNA aminoacylation were measured at 25°C in 0.1 mL of 20 mM Imidazole-HCl buffer (pH 7.5), 100 mM KCl, 0.5 mM DTT, 12 mM MgCl_2_, 2 mM ATP, 180 µM ^14^C-labeled lysine (NEN; 16.66 Ci/mol), and saturating amounts of global yeast tRNA (30). The incubation mixture contained catalytic amounts (2 nM) of mLysRS appropriately diluted in 10 mM Tris-HCl (pH 7.5), 10 mM 2-mercaptoethanol, containing bovine serum albumin at 4 mg/mL. Inhibitors were added at final concentrations ranging from 3.9 to 1,000 µM. One unit of activity is the amount of enzyme producing 1 nmol of lysine-tRNA^Lys^/min, at 25°C.

## SUPPLEMENTAL MATERIAL

Supplemental figures mbio.02087-25-s0001.pdf Fig. S1 to S6.

ASM does not own the copyrights to Supplemental Material that may be linked to, or accessed through, an article. The authors have granted ASM a non-exclusive, world-wide license to publish the Supplemental Material files. Please contact the corresponding author directly for reuse.
