# Supplementary material for: Inhibition of HIV-1 replication by primer RNA packaging inhibitors
Source: mBio. 2025 Oct 23;16(11):e02087-25. doi: 10.1128/mbio.02087-25 (PMC12607897; doi:10.1128/mbio.02087-25)
Supplement: Supplemental figures — Fig. S1 to S6. [file mbio.02087-25-s0001.pdf]

Supplementary Material to :

Inhibition of HIV-1 replication by primer RNA packaging inhibitors.

Marc Mirande*, Frédéric Subra, Clémence Richetta, Eric Deprez and Olivier Delelis

Laboratoire de Biologie et Pharmacologie Appliquée (LBPA), UMR 8113 CNRS, ENS Paris-Saclay, Université Paris-Saclay, 91190 Gif-sur-Yvette, France

*Corresponding author.

email: marc.mirande@ens-paris-saclay.fr

Supplementary Figure Legends

Fig. S1. Determination of half-maximal inhibitory concentration (IC50) of the drugs in the mLysRS:IN-CTD222 and mLysRS:Pol binding assays. The binding of mLysRS to IN-CTD222 (left) or Pol (right) was monitored in the HTRF assay in the presence of increasing concentration of the drugs (10 to 2000 µM). Experimental values (symbols) were fit (curves) to the equation Y = Ymin + ((Ymax-Ymin)/(1+(X/IC50)^n)), where n is the Hill coefficient. The IC50 of the drugs in the binding assays and the associated standard deviations (n=3) are indicated. The dimer concentration of mLysRS is 1.5 nM, the monomer concentrations of IN-CTD222 and Pol are 1.2 µM and 20 nM, respectively.

Fig. S2. Chemical structures and IC50 of lacidipine and cilnidipine derivatives carrying a dihydropyridine ring. IC50 were determined as described in the legend of figure 1.

Fig. S3. Effect of the inhibitors on HIV-1 replication and cell toxicity. MT4 cells were infected with NL4-3-GFP virus. Two days after infection, inhibitors were added to the culture medium at final concentrations of 1, 3.3 or 10 µM. The final concentration of DMSO was 0.1 %. Five days after infection, (a) GFP expression was quantified by flow cytometry, and (b) cell viability was determined by the MTT assay. NInf corresponds to non-infected cells. Three independent experiments were performed and standard deviations are shown.

Fig. S4. Raw data of GFP-negative and GFP-positive cells recovered after 72 h incubation in the presence of 10 µM of the drugs, as determined by FACS analysis.

Fig. S5. Effect of inhibitors on mLysRS activity. The tRNA-aminoacylation activity of mLysRS was determined in the presence of increasing concentrations of the drugs. Two independent experiments were performed and standard deviations are shown.

Fig. 6. Raw data of quantification of minus-strand strong stop viral DNA and viral RNA by digital PCR from viruses recovered after 72 h incubation in the presence of 10 µM of the drugs.

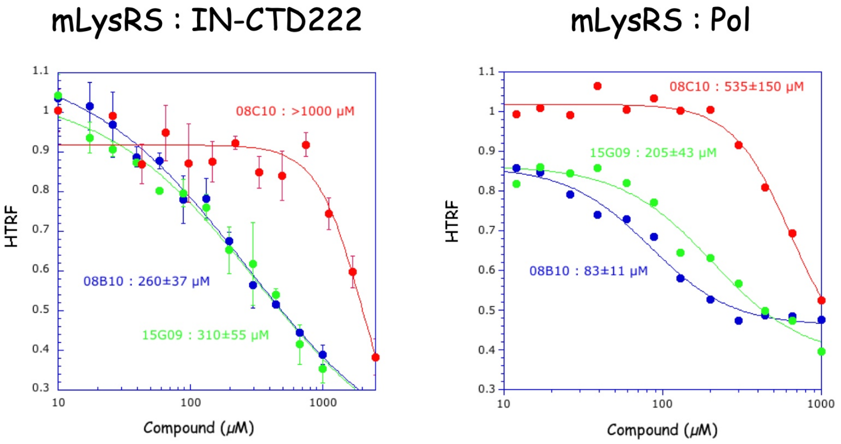
mLysRS : IN-CTD222
08C10 : >1000 µM
08B10 : 260±37 µM
15G09 : 310±55 µM
HTRF
Compound (µM)
mLysRS : Pol
08C10 : 535±150 µM
15G09 : 205±43 µM
08B10 : 83±11 µM
HTRF
Compound (µM)

Figure S1

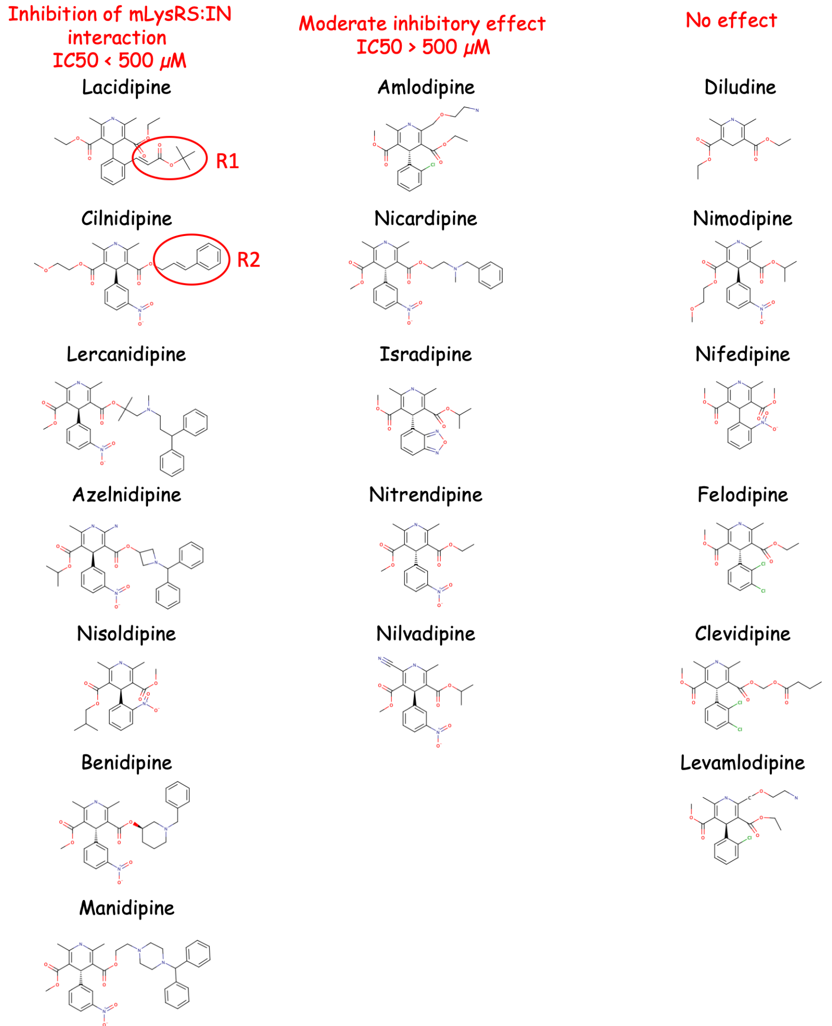
Inhibition of mLysRS:IN interaction
IC50 < 500 µM
Moderate inhibitory effect
IC50 > 500 µM
No effect
Lacidipine
R1
Amlodipine
Diludine
Cilnidipine
R2
Nicardipine
Nimodipine
Lercanidipine
Isradipine
Nifedipine
Azelnidipine
Nitrendipine
Felodipine
Nisoldipine
Nilvadipine
Clevidipine
Benidipine
Levamlodipine
Manidipine

Figure S2

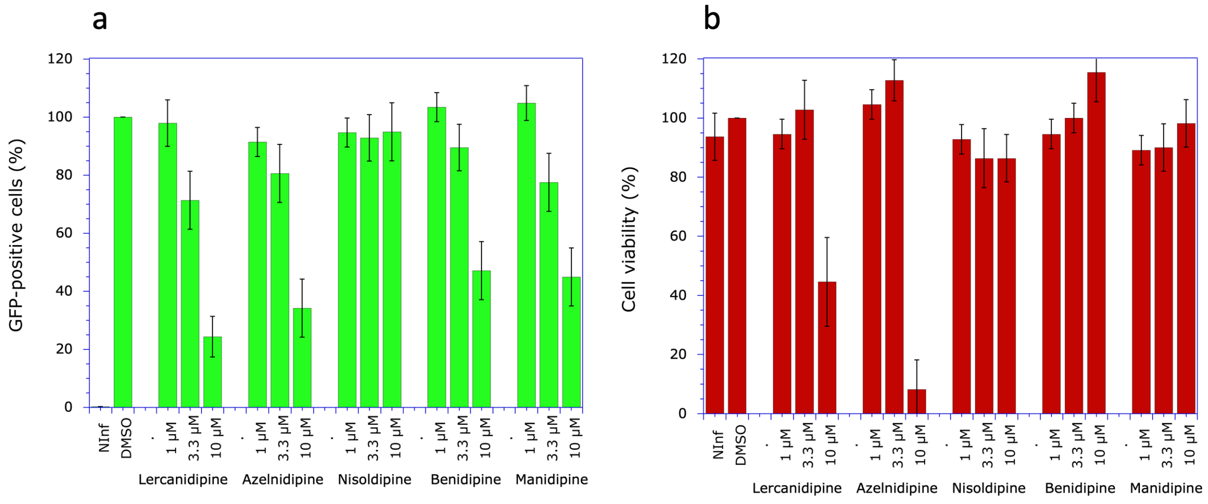
a
GFP-positive cells (%)
120
100
80
60
40
20
0
NInf
DMSO
1 µM
3.3 µM
10 µM
Lercanidipine
Azelnidipine
Nisoldipine
Benidipine
Manidipine
b
Cell viability (%)

Figure S3

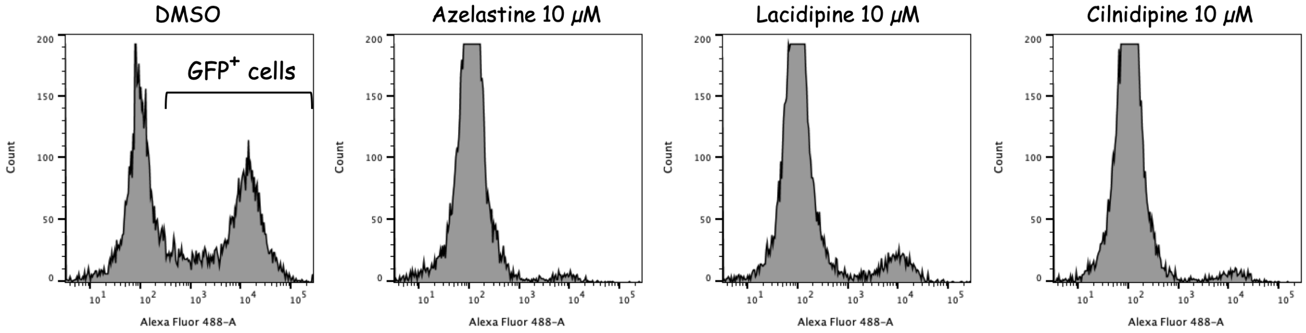
DMSO
Azelastine 10 μM
Lacidipine 10 μM
Cilnidipine 10 μM
GFP+ cells
Count
Alexa Fluor 488-A

Figure S4

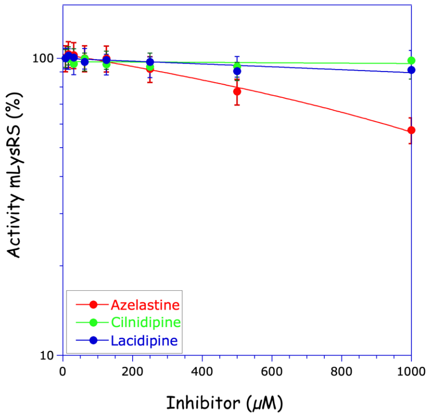

Activity mLysRS (%)
100
10
0
200
400
600
800
1000
Inhibitor (μM)
Azelastine
Cilnidipine
Lacidipine

Figure S5

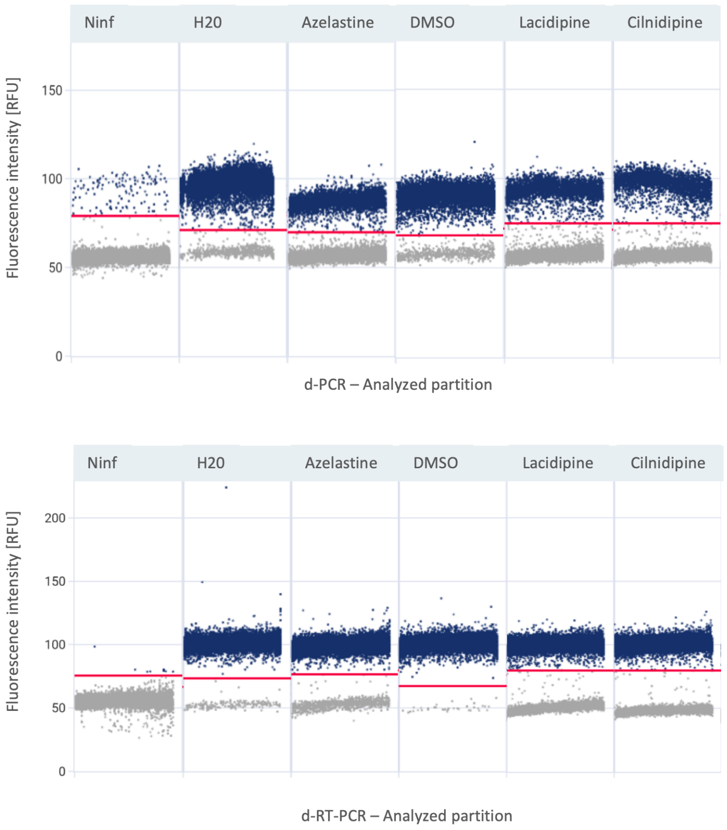
Ninf
H20
Azelastine
DMSO
Lacidipine
Cilnidipine
Fluorescence intensity [RFU]
150
100
50
0
d-PCR – Analyzed partition
Ninf
H20
Azelastine
DMSO
Lacidipine
Cilnidipine
Fluorescence intensity [RFU]
200
150
100
50
0
d-RT-PCR – Analyzed partition

Figure S6
